# Scale-up of Multidrug-Resistant Tuberculosis Laboratory Services, Peru

**DOI:** 10.3201/eid1405.070721

**Published:** 2008-05

**Authors:** Sonya S. Shin, Martin Yagui, Luis Ascencios, Gloria Yale, Carmen Suarez, Neyda Quispe, Cesar Bonilla, Joaquin Blaya, Allison Taylor, Carmen Contreras, Peter Cegielski

**Affiliations:** * Brigham and Women’s Hospital, Boston, Massachusetts, USA; † Instituto Nacional de Salud, Lima, Peru; ‡ Programa de Control de Tuberculosis, Lima, Peru; § Partners in Health, Boston, Massachusetts, USA; ¶ Centers for Disease Control and Prevention, Atlanta, Georgia, USA; # Socios en Salud, Lima, Peru

**Keywords:** Tuberculosis, multidrug resistance, laboratories, clinical laboratory information systems, health facilities, Peru, health plan implementation, regional health planning, resource-poor settings, perspective

## Abstract

Over the past 10 years, the Peruvian National Tuberculosis (TB) Program, the National Reference Laboratory (NRL), Socios en Salud, and US partners have worked to strengthen the national TB laboratory network to support treatment of multidrug-resistant TB. We review key lessons of this experience. The preparation phase involved establishing criteria for drug susceptibility testing (DST), selecting appropriate DST methods, projecting the quantity of DST and culture to ensure adequate supplies, creating biosafe laboratory facilities for DST, training laboratory personnel on methods, and validating DST methods at the NRL. Implementation involved training providers on DST indications, validating conventional and rapid first-line DST methods at district laboratories, and eliminating additional delays in specimen transport and result reporting. Monitoring included ongoing quality control and quality assurance procedures. Hurdles included logistics, coordinating with policy, competing interests, changing personnel, communications, and evaluation. Operational research guided laboratory scale-up and identified barriers to effective capacity building.

## Abstract 2

One-sentence summary for table of contents: Strategic design and implementation of these services is feasible in resource-poor settings.

Heightened awareness of the global threat of tuberculosis (TB) has been spurred, in part, by the widespread prevalence of drug-resistant strains (*1*). Extensively drug-resistant TB (XDR TB) is associated with high death rates among patients co-infected with HIV and has led to renewed efforts to strengthen TB control (*2*,*3*) Program managers and policy makers face the urgent task of quickly scaling-up comprehensive TB programs, often in settings with minimal infrastructure. Although daunting, the task appears feasible in light of favorable early treatment outcomes for multidrug-resistant TB (MDR TB) treatment programs, the growing cadre of technical experts, consensus on TB and MDR TB management (*4*), and availability of global resources to fund programs (*5*,*6*).

From 1996 through 2005 in Peru, a consortium of institutions implemented one of the most comprehensive national MDR TB treatment programs in the world. One component of this effort was the Laboratory Improvement Project, which was charged with scaling-up laboratory services to support MDR TB treatment. We encountered many lessons in expanding laboratory access to quality TB culture and drug susceptibility testing (DST). We summarize the key lessons that may be relevant for other settings where MDR TB treatment is being planned or implemented.

## Background

TB incidence in Peru is among the highest in Latin America, at 108.2/100,000 persons in 2005 (Table 1) (*7*). In the densely populated periphery of Lima, where half of all national cases are detected, the risk for infection with *Mycobacterium tuberculosis* may be among the highest recently documented (*8*–*10*). Rates of MDR TB are also high, with a national prevalence of 3% among patients never treated for TB and 12.3% among previously treated patients (*11*). During 1990–2000, Peru implemented a model program based on the World Health Organization (WHO)–endorsed strategy of directly observed treatment, short course (DOTS) (*12*). Massive use of sputum smear microscopy and standardized first-line treatment resulted in effective case detection and cure, with an overall decrease in TB incidence by the end of the decade (*13*). During that period, however, the rates of MDR TB increased (*14*).

**Table 1:** HIV and tuberculosis (TB), Peru, 2005*

Characteristic	Value
Total population	28,300,000
Population in Lima	7,300,000
Average life expectancy, y	69
Infant mortality rate	31/100,000 live births
GDP per capita	$2,500
Population living in poverty	54%
National HIV prevalence	0.6%
Estimated no. HIV positive	60,000–80,000
No. receiving HIV therapy	9,157
TB incidence	108/100,000
MDR TB in new patients	3%
MDR TB in previously treated patients	12.3%
TB in HIV patients	≈30%
HIV in TB patients	≈3%
MDR TB in co-infected patients	30%–47%
Mortality rate among co-infected patients†	<38%
Mortality rate among MDR TB–HIV patients	<57%

*GDP, gross domestic product; MDR TB, multidrug-resistant TB. †Co-infected with HIV and TB but not necessarily MDR-TB.

Because DOTS alone was insufficient to control ongoing transmission of drug-resistant strains (*15*), Partners in Health (PIH), Harvard University, Massachusetts State Laboratory Institute (MSLI), Socios en Salud, the Peruvian National Tuberculosis Control Program (NTP), and the Peruvian National Institute of Health (INS) initiated a collaborative MDR TB treatment effort in 1996 (*16*). Principles included individualized MDR TB treatment and monthly culture to monitor treatment response. Community health promoters provided direct observation of all doses given outside health clinic hours. In 1997, the NTP implemented a standardized MDR TB treatment regimen, which achieved cure rates <50% (*17*). Although protocols changed over time, treatment failures, defaulters, and relapses after first-line treatment were generally referred for standardized MDR TB therapy. Those patients whose standardized treatments failed were, in turn, referred for individualized treatment.

### Expansion of Laboratory Capacity, 1996–2000

When we began this project, 1 level III laboratory, the National TB Reference Laboratory, performed DST on first-line drugs; 57 level II laboratories performed mycobacterial culture, and ≈1,000 level I laboratories had smear microscopy capacity (Table 2). Because DST on second-line drugs was not available in Peru, isolates were initially sent to the MSLI until local capacity could be established.

**Table 2:** Baseline laboratory capacity for diagnosis of tuberculosis, Peru, 1996–2000*

Activity	Validation or quality control procedures	No. establishments	No. performed/year
Smear microscopy	Quality control of all AFB+ and 10% of AFB– results each trimester at regional level of laboratories	987	1,164,198
Mycobacterial culture	Once a year, quality control of media culture	57	48,346
Drug susceptibility testing	External quality control in INPPAZ	1	1,045

*AFB, acid-fast bacilli; INPPAZ, Instituto Panamericano de Protección de Alimentos y Zoonosis.

**Figure 1:**
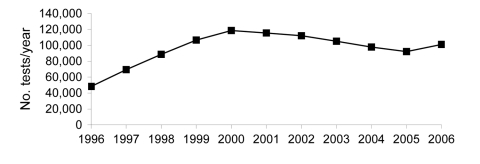
Mycobacterial cultures performed in Peru, by year.

**Figure 2:**
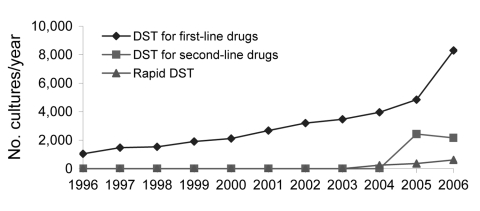
Drug susceptibility testing (DST) performed in Peru, by method and year.

As the MDR TB treatment program expanded in absolute numbers and geographic coverage, so too did demand for laboratory services. From 1996 through 2000, the number of mycobacterial cultures and DSTs performed yearly more than doubled (Figures 1, 2). The process of program scale-up posed additional challenges in patient management, information systems, drug procurement, and regional implementation. Responding to these needs, the Bill & Melinda Gates Foundation awarded a grant for $45 million in 2000 to establish a consortium called PARTNERS, whose principal task was to achieve national coverage of MDR TB treatment in Peru and replicate this project elsewhere. Several key institutions were added to the initial group of collaborators: WHO, the Centers for Diseases Control and Prevention (CDC), and the Task Force for Child Survival and Development. Within the PARTNERS consortium, the Laboratory Improvement Project was established with specialists from MSLI, CDC, Harvard University, PIH, and INS.

### Strategy to Scale-up Laboratory Services

NTP norms for DST indications have evolved over the past 10 years. This heterogeneous and dynamic process provided lessons on matching the choice of DST to programmatic strategies (Table 3). Salient aspects guiding laboratory strategies include the choice of standardized versus individualized treatment, criteria for performing DST, rates of HIV and resistance to second-line drugs, and empiric management while awaiting results.

**Table 3:** Optimal DST characteristics depending on MDR TB management strategy, Peru*

Programmatic and epidemiologic features	Optimal DST characteristics
Standardized versus individualized regimens	
Standardized regimens for MDR based on regional resistance patterns	Centralized, complete DST (i.e., first- and second-line drugs) of representative samples to guide standardized treatment regimen; turnaround time less important
Individualized regimens	Rapid, point-of-care DST optimal to accommodate high demand and minimize turnaround time. Semi-individualized regimens may be constructed if only DST to first-line drugs performed.
Who is tested for DST?	
Narrow DST indications (e.g., treatment failures only)	High pretest probability for MDR TB; therefore, optimal to perform DST to first- and second-line drugs to guide regimen design
Moderate DST indications (e.g., healthcare worker, smear-positive in second month of DOTS)	Rapid DST to first-line drugs to screen MDR TB versus non–MDR TB. If individualized treatment, drug-resistant samples may be referred for complete DST. Sensitivity may be more important than specificity because of greatest illness from failing to start appropriate treatment in patients with drug resistance.
Universal DST	Rapid DST to first-line drugs to screen MDR TB versus non–MDR TB. Rapid point-of-care testing (decentralized) optimal. If individualized treatment, drug-resistant samples may be referred for complete DST. Sensitivity may be more important than specificity.
Epidemiologic features	
Patients with smear-negative disease (e.g., HIV, children)	Direct DST by using liquid medium or indirect DST after culture by liquid medium. Rapid turnaround time important given high illness rates in these risk groups.
High rates of resistance to second-line drugs (XDR TB)	Complete DST if high rates of resistance to second-line drugs, including XDR. If limited resources, DST to first-line drugs plus key second-line drugs (e.g., quinolone, kanamycin) to enable identification of XDR TB cases.
Management while awaiting DST results	
Empiric first-line regimen	Greater risk for inadequate treatment of MDR TB cases; rapid testing more important
Empiric MDR TB regimen	Less risk for inadequate treatment of MDR TB cases, excess cost and toxicity for non–MDR TB cases. Complete DST results permit adjustment of empiric MDR TB therapy.

*DST, drug susceptibility testing; MDR TB, multidrug-resistant tuberculosis; XDR TB, extensively drug-resistant TB; DOTS, directly observed treatment, short course.

On the basis of projected numbers, DST needs would not be met unless DST on first-line drugs was decentralized to regional laboratories in areas with high rates of TB and MDR TB. In choosing methods for decentralized DST, the INS matched method features with available resources in regional laboratories (Table 4). The need for a rapid DST method was clear. Given that it took an average of almost 5 months to obtain results from a conventional DST performed in Peru (*18*), physicians often had to make treatment decisions empirically. Once results did arrive, they were no longer accurate because patients had been exposed to additional drugs in the interim, to which amplified resistance could have occurred. Rapid DST implemented at the decentralized level would be the most effective way of providing timely results and decompressing the central bottleneck of DST demand.

**Table 4:** Considerations for decentralized drug susceptibility testing (DST) capacity for first-line drugs, Peru

Criterion	Ideal situation
Drugs to test	First-line DST; isoniazid and rifampin most important because empiric treatment regimen and further DST may follow
Reproducibility	Because drug-resistant samples identified by regional DST, then referred to National Reference Laboratory for DST to second-line drugs, sensitivity most important
Sample source	Direct method optimal for processing at local health clinic to minimize turnaround time
Cost per sample	Low cost
Time to obtain result	Rapid
Technical demand	Less technically demanding, less processing time
Biologic safety risk	Low biosecurity risk
Required equipment	Limited additional equipment (refridgerated centrifuge) procured and maintained in local site
Reagents and supplies	Commonly used reagents and supplies available through local vendors is preferable

**Figure 3:**
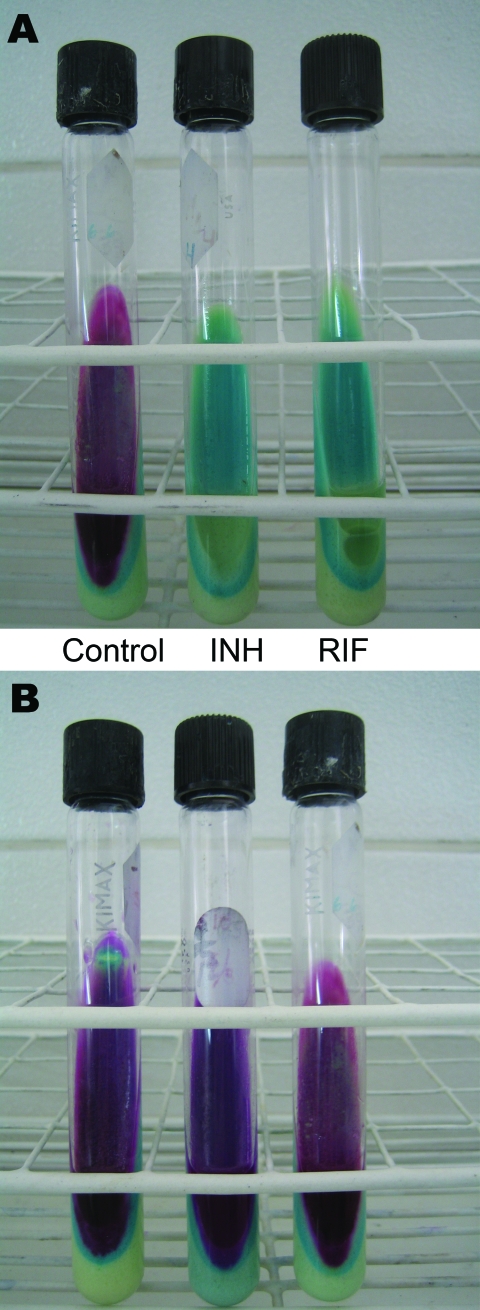
Description and costs of the direct Griess method in Peru. A) Pan-susceptible *Mycobacterium tuberculosis* isolate. B) *M. tuberculosis* isolate resistant to isoniazid (INH) and rifampin (RIF). The left (control) tube in panel A and all tubes in panel B indicate mycobacterial growth. The costs of the test are US $5.30 per sample, including personnel, materials (items that can be reused), and supplies (reagents and consumable items), and US $4.80 per sample, including materials and supplies.

The INS decided that rapid DST should serve as an initial screening test. By quickly identifying resistance to isoniazid and rifampin, isolates with drug resistance could be sent to INS for full DST while standardized MDR TB treatment was started. With input from MSLI, the INS chose the Griess method. This method is a rapid colorimetric method that uses Lowenstein-Jensen (LJ) medium prepared with antimicrobial drugs (Figure 3) (*19*). Previously the method was validated as an indirect method; however, INS opted to implement it as a direct method, i.e., it is performed directly with sputum. INS validation of this method yielded sensitivities and specificities of 99% and 100% to isoniazid and 94% and 100% to rifampin (*20*). Attributes of the Griess method are accuracy, fast turnaround time (21 days), minimal additional equipment needs, inexpensive materials and reagents, and reproducibility in laboratories proficient in mycobacterial culture.

On the basis of this rationale, the following plan was developed. Second-line DST (agar plate proportions method) would be implemented in the INS. Conventional first-line DST (proportions method, indirect variation by LJ medium) would be performed at regional laboratories. Direct Griess method would be performed at regional laboratories; and the indirect BACTEC-460 system (Becton Dickinson, Franklin Lakes, NJ, USA) for first-line drugs would be implemented at INS for high-risk patients, including healthcare workers, HIV-positive patients, and pediatric patients.

Another priority was reducing the overall turnaround time of laboratory data, defined as the time when the patient is first identified at risk for MDR TB to the time that this determination has an effect on patient care. Before DST decentralization, we conducted an assessment of turnaround times in 2 health districts and confirmed that laboratory efficiency, including decentralization of DST and implementation of rapid methods, would have limited effect if pre- and post-DST processing delays were not addressed (*18*). These delays included specimen transport, specimen processing, dissemination of results to the health center, and scheduling of clinical evaluation once results were obtained. Of 924 samples processed over 16 months, the median turnaround time was 147 days; only 81 days were caused by DST processing. On the basis of these data, we worked with leaders at national and regional levels to develop and implement strategies to reduce delays (Table 5).

**Table 5:** Strategies to reduce turnaround time of culture and DST, Peru*

Step	Median baseline turnaround time, d	Strategies used	Goal turnaround time, d
From time DST processed to DST result at INS	81	Decentralize conventional and rapid DST methods	21
From receipt of DST result at intermediate laboratory to receipt of DST result at health establishment	6	Implement laboratory information system linking health centers, regional and national laboratories; improve transport of samples from health centers to regional laboratories	1
From receipt of DST result at health establishment to patient reevaluation with DST result	33	Train local providers to improve identification and referral of patients in need of MDR TB treatment; increase frequency of MDR TB treatment approval meetings; create new national culture/DST request form with DST indicators	7

*DST, drug susceptibility testing; INS, Instituto Nacional de Salud; MDR TB, multidrug-resistant tuberculosis.

The overall strategy for laboratory scale-up comprised the following activities. First, establish clear criteria for performing DST. Second, select DST methods for use within the TB program and indications for each method. Third, decentralize first-line DST to 7 regional laboratories. Fourth, project the quantity of DST and cultures and ensure adequate supplies. Fifth, create biosafe laboratory facilities for DST. Sixth, train laboratory personnel on new methods. Seventh, train healthcare providers and level I laboratory personnel on DST indications. Eighth, validate DST methods, first in the INS and then at each implementing site. Ninth, establish and enact quality control and quality assurance protocols. Tenth, eliminate additional delays in specimen transport and result reporting. These strategies were used and modified in 3 phases of scale-up: preparation, implementation, and monitoring.

### Preparation Phase

Key elements of the preparation phase were mobilizing political commitment (i.e., agreeing upon the strategic plan, obtaining adequate financial and human resources, and formalizing collaborations and the respective roles of different, competing and cooperating, institutions); establishing adequate laboratory infrastructure; and forming a skilled workforce. A needs assessment performed early in the project identified the need for documented biologic safety cabinet (BSC) certification and maintenance and repair of BSCs throughout the TB laboratory network. Because Peru had no trained personnel who could certify BSCs, a training program was developed and delivered with the help of MSLI and the Eagleson Institute in Sanford, Maine. The trained certifiers then certified and repaired BSCs for the TB laboratory network.

To proceed with decentralization efforts, INS contacted directors of regional laboratories. Only 1 of the laboratories met minimal space and biologic requirements to safely perform DST. The remaining 6 laboratories were asked to submit a proposal for laboratory renovations; only 3 were able to respond in a timely fashion. We explored why the other 3 laboratories did not respond and found that the administrative time and technical expertise required to elaborate a proposal was often not within the capacity of district and laboratory leaders.

We supported 2 laboratory renovations and discovered that substantial time and resources were required to complete this process. Producing detailed and thorough technical proposals required substantial input from a range of experts, including architects; building, sanitary and electrical engineers; and construction companies. We identified experts with interest and competence in designing TB health facilities and encouraged collaboration by team, with technical assistance from an engineer experienced in TB infection control at CDC. Cultivating such a team with specialized knowledge in TB infrastructure has proven to be an asset for Peru. This team has since worked on other projects to renovate TB clinics and laboratories.

Once elaborated, the proposals then required approval by the governmental institution responsible for approving renovations and construction of public health facilities. Construction for both projects was delayed by an average of 6 months because of these administrative requirements. District and laboratory leaders played an important role by making frequent inquiries into the status of the approval process. In the meantime, we purchased necessary equipment, materials, and supplies.

Another step to expand DST capacity was the training and validation process for each DST method. MSLI trained INS in DST to second-line drugs by the agar plate proportion method; validation was completed in 2005. Concomitantly, INS trained regional laboratory personnel in DST of first-line line drugs, by the LJ medium proportions method. To initiate rapid DST, the Griess method was validated first at INS; then personnel from each implementing laboratory were trained in the method. Both conventional DST and rapid DST were validated at the regional laboratories. Samples were collected under program conditions. DST was performed by trained personnel in the regional laboratories. These same strains were then sent to INS for validation.

INS also validated BACTEC against LJ medium proportions and sped the process by performing BACTEC culture followed by indirect BACTEC DST on first-line line drugs. Validation was done for the AccuProbe method (Gen-Probe, Inc., San Diego, CA, USA) to identify *M*. *tuberculosis* and *M*. *avium* complex. Finally, INS leaders developed standard operating procedures, including protocols for all laboratory methods, biosafety and equipment standards, and quality assurance and quality control procedures.

Other activities during the preparation stage were aimed at reducing turnaround time. We developed and piloted an electronic laboratory information system connecting INS, regional laboratories, and health centers to provide health personnel (physicians, nurses, and laboratory technicians) with real-time access to culture and DST results. To support the system, we worked with health district leaders to provide Internet access, computers, and Web access points at health centers (*21*). We also purchased 2 automobiles to aid in specimen transport. At the administrative level, NTP increased the frequency of MDR TB treatment–approval meetings to reduce the bottleneck of cases pending approval for initiation of MDR TB treatment.

### Implementation Phase

After successful completion of validation procedures in regional laboratories, DST was incorporated into programmatic services. Aggregate data on DST results were reviewed by each laboratory on a monthly basis to monitor rates of contamination, culture growth, and drug resistance. INS supervisors made frequent visits to these laboratories to monitor performance and troubleshoot any challenges. For instance, when low rates of culture growth were observed among acid-fast bacilli smear-positive samples, smear microscopy slides from these samples were reviewed by a biologist and decontamination protocols were reviewed. During this period, we simultaneously trained healthcare personnel in workshops and one-on-one interactions. Laboratory and TB program directors led workshops to review programmatic norms for soliciting each DST method and to explain the performance and characteristics of each method. Health workers were also trained to use the laboratory information system. Regional administrators trained providers in patient confidentiality and established a plan for sustained Internet access and computer maintenance after the pilot phase of the information system. We secured the commitment of health center directors to guarantee that TB personnel would have access to the computers during designated hours because computers were rarely placed in the TB services areas to reduce the risk for theft and vandalism.

### Monitoring Phase

Sustainable laboratory infrastructure depends on administrative commitment and monitoring laboratory performance quality. Throughout the entire planning and implementation stages, MSLI provided training to INS and regional laboratories in basic and method-specific quality control/quality assurance.

The appropriate use of DSTs and culture data by healthcare workers also required ongoing evaluation. Preliminary data demonstrate that despite the reinforcement of NTP norms, health personnel often failed to adhere to NTP norms for DST (*22*). Approximately 50% of DSTs in 2005 in Lima were requested for patients without an indication for testing by NTP norms. Of DSTs not meeting NTP norms, ≈28% of these were for patients who had MDR TB compared with 32.5% among those with NTP criteria. These findings support the need for broadened indications for DST. Monitoring laboratory and programmatic performance was not effective unless these data were fed back to healthcare personnel. An example is a series of reports generated by the information system and provided to laboratory and regional TB program directors (Table 6) (*23*,*24*).

**Table 6:** Automated reports generated by tuberculosis (TB) laboratory information system, Peru*

Report	Informed	Purpose	Type of access†
Frequency of information system access by healthcare center personnel	Regional laboratory and TB director	Maintain frequent use of information system to access real-time laboratory data	Monthly report prepared by data administrator
No. laboratory results entered at regional laboratory	Regional laboratory and TB director	Identify delays in data entry	Monthly report prepared by data administrator
No. laboratory results verified and released to providers	Regional laboratory and TB director	Identify delays lags in result verification	Monthly report prepared by data administrator
DST results for any specified period grouped by every variable in request form	Regional and INS laboratory director	Report and identify trends in laboratory performance	Constant
Culture results for any specified period grouped by every variable in request form	Regional and INS laboratory director	Report and identify trends in laboratory performance	Constant
DST and cultures in process too long, DST missing reception date, DSTs needed to be entered into system, duplicate tests	Regional and INS laboratory director	Quality control	Constant
Rate of culture contamination; rate of negative culture growth from smear-positive specimens	Regional and INS laboratory director	Identify trends in laboratory performance	Constant
Persons with a positive culture for any specified date	Regional and INS laboratory director	Reporting to regional TB program	Constant
Persons with new DST or culture results	Healthcare center personnel	Minimize turnaround time of laboratory results	Constant and email notification
Tests that are in process and the number of days in process	Healthcare center personnel	Inform personnel of when to expect results	Constant

*DST, drug susceptibility testing; INS, Instituto Nacional de Salud. †Constant access indicates that laboratory users could view this information in the system at any time. Some reports let the user specify the start and end dates.

TB management protocols, such as DST indications and optimal DST methods, are dynamic; they must respond to changes in regional epidemiology as well as the availability of resources. For example, decentralization of DST resulted in an increased demand for DST because of increased awareness of MDR TB and availability of testing. Additionally, health professionals and patients perceived the benefit of rapid, real-time laboratory data. This increase in demand is an example of how our ongoing monitoring and evaluation could be applied to reassess the use and capacity of laboratory services. Our preliminary data of adherence to NTP indications for DST (*22*) and rates of MDR TB among risk groups (*25*) have helped inform modifications of NTP policy. The experience thus far in matching the appropriate DST methods to NTP norms should enable a rational application and operational assessment of promising new DST methods (*26*). Without adequately quantifying and responding to an increase in DST demand, laboratory operations may become bottlenecked, and excessive demand on limited personnel could result in deviations from laboratory protocols and a decrease in laboratory performance. Figures 1 and 2 reflect the level of laboratory expansion in Peru as of 2006, which demonstrates the trajectory of scale-up, not only in terms of DST, but for culture as well.

## Lessons Learned

TB programs faced with incorporating MDR TB treatment must often expand laboratory infrastructure far beyond existing capacity. Although laboratory improvement efforts in Peru have taken a decade to accomplish and are still evolving, several key lessons can be distilled from our experience.

### Responding in Time and Stepwise, Overlapping Efforts to Prevent Delays

The introduction and decentralization of DST and culture capacity can involve a wide range of activities, ranging from obtaining permits from national authorities to purchasing automobiles to streamline specimen transport. Attention to detail, the dedication of human resources to push these activities along, and parallel planning and coordination of activities can receive inadequate priority among program planners. Although these logistics can be painfully mundane, they are often the greatest obstacles, thus indirectly causing the most serious illness due to excessive delays. The recent outbreak of XDR TB among HIV-positive populations in KwaZulu-Natal, South Africa, demonstrates the need to scale-up laboratory services in a timely but correct manner (*27*).

### Coordination of National Reference Laboratory and National TB Programs

Political commitment must include stable leadership; a strong central, coordinating unit; and a working relationship between TB laboratories and a TB program (*28*). The importance of coordinating laboratory and programmatic efforts may seem obvious but cannot be overstated. Within the DOTS model, smear microscopy can be performed at health centers with local coordination with TB services. In contrast, MDR TB treatment requires more complex methods (culture, DST) and is usually performed and overseen at a central site. Strategies must be informed by NTP policy and vice versa. Coordination must persist because the needs of a TB program will likely change over time.

### Importance of Operational Research

Our experience in Peru was informed by our operational research. The profile of a DST method and its characteristics, when first validated in a local laboratory, may be different from its performance, strengths, and weakness when it is operating under actual program conditions. Operational assessment of a laboratory method or strategy is the sole means of understanding its effectiveness when considered within the larger context of how the method is used, associated complexities or challenges in its implementation, the mitigation of its effect caused by other system delays, and other factors. If tools to monitor laboratory performance are incorporated into information and reporting systems at the outset, effective operational research can be conducted with minimal additional resources, coupled with ongoing feedback, to create a sustainable laboratory system.
